# Supplementary material for: Xylose and shikimate transporters facilitates microbial consortium as a chassis for benzylisoquinoline alkaloid production
Source: Nat Commun. 2023 Nov 28;14:7797. doi: 10.1038/s41467-023-43049-w (PMC10684500; doi:10.1038/s41467-023-43049-w)
Supplement: Supplementary file 1 — Supplementary Information [file 41467_2023_43049_MOESM1_ESM.pdf]

Xylose and shikimate transporters facilitates microbial consortium as a chassis for benzylisoquinoline alkaloid production

Gao *et al*.

Supplementary Table 1. The differential expression of the genes involved in xylose uptake and utilization in *Scheffersomyces stipitis* under various sugar conditions. RNA-seq was performed to analyze cells growing in different sugars following the protocol reported earlier[1]. The cultures were sampled at 15 h, 48 h, and 72 h, which respectively corresponded to the 'prior to glucose depletion', 'right after glucose depletion', and 'post-glucose depletion' stages in the mixed-sugar culture. The ratio of the average sequencing reads for each gene under three different growing conditions (i.e., X/G and X/GX) was calculated. X, YPA+15 g/L xylose; G, YPA+35 g/L glucose; GX, YPA+35 g/L glucose and 15 g/L xylose.

**Carbon source**	**Sampling time**	**Target genes**					
		XYL1	***XYL2***	***XYL3***	***TKT1***	***TAL1***	***XUT1***
X/G	15 h	34.7	7.3	56.3	3.8	5.6	16.7
	48 h	14.0	2.2	5.3	1.8	2.1	18.8
	72 h	0.2	0.2	0.3	0.5	0.4	1.2
X/GX	15 h	21.3	7.0	9.7	4.0	4.2	15.0
	48 h	1.0	0.7	0.6	1.0	0.8	15.4
	72 h	0.007	0.03	0.02	0.3	0.1	0.09

Supplementary Table 2. Xylose assimilating capability of CUG clade yeasts, and the maximal identities of the corresponding homologs to SpXut1.

Microorganism	Xylose-assimilating ability	Maximal identity (%)
Spassaspora passalidarum	yes	100
Scheffersomyces stipitis	yes	79
Candida albicans	yes	67
Candida dubliniensis	only 5% assimilate xylose	67
Candida maltosa	yes	67
Candida tropicalis	yes	66
Candida tenuis	yes	56
Candida guilliermondii	yes	41
Candida auris	no	39
Debaryomyces hansenii	yes	37
Lodderomyces elongisporus	yes	37
Candida tanzawaensis	no	36
Hyphopichia burtonii	yes (weak)	34
Canada lusitaniae	yes	34
Metschnikowia bicuspidata	some yes, some no	34
Candida orthopsilosis	yes	32
Candida parapsilosis	yes	32
Metschnikowia fructicola	yes (weak)	n/a
Meyerozyma caribbica	yes	n/a
Spassaspora arborariae	yes	n/a
Candida oleophila	yes	n/a
Candida sojae	yes	n/a

Note: n/a, no genome sequence is available, or no significant similarity was found. All data of xylose-assimilating ability were collected from National Collection of Yeast Cultures (https://catalogue.ncyc.co.uk/) except *Candida auris*[2] and *Spassaspora arborariae*[3], which were individually reported.

Supplementary Table 3. Eight quinate permeases for shikimate uptake assay. The query sequence was AnQut1.

Transporters	NCBI gene ID	Number of intron(s)	NCBI accession number	Protein length (aa)	Identity to AnQut1 (%)	Similarity to AnQut1 (%)
AnQut1	4982700	1	XP_001392502.1	535	100	100
AnQut2	4979277	2	XP_001399921.1	536	64	78
AnQut3	4987285	2	XP_001401052.1	518	36	56
AnQut4	4988028	3	XP_001396962.2	543	33	53
AnQut5	4985506	4	XP_001395243.2	534	34	51
AnQut6	4989730	3	XP_001398629.1	553	31	52
AnQut7	4985176	11	XP_001394918.2	566	33	50
AnQut8	4978462	3	XP_001388526.2	532	33	52

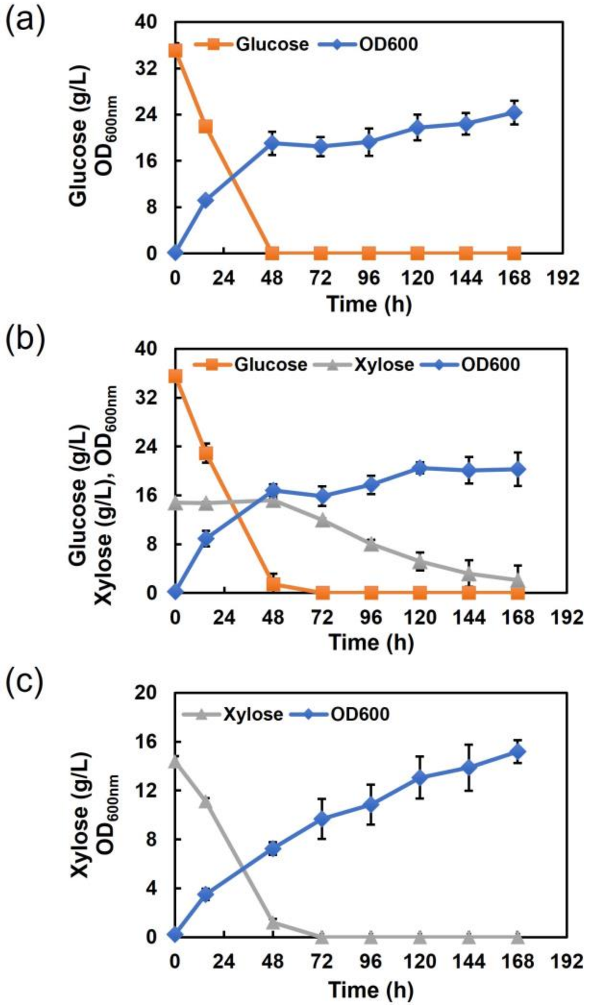

Supplementary Figure 1. The growth and sugar consumption profiles of S. stipitis cultured in YPA plus glucose, xylose, or both sugars. *S. stipitis* cultured in YPA plus 35 g/L glucose (a), YPA plus 35g/L glucose and 15 g/L xylose (b), and YPA plus 15 g/L xylose (c). Initial cell density was at ~0.2. Data are presented as mean ± S.D., *n* = 3 per group. This figure was borrowed from our previous publication to demonstrate the level of CCR in *S. stipitis*[1]. Source data are provided as a Source Data file.

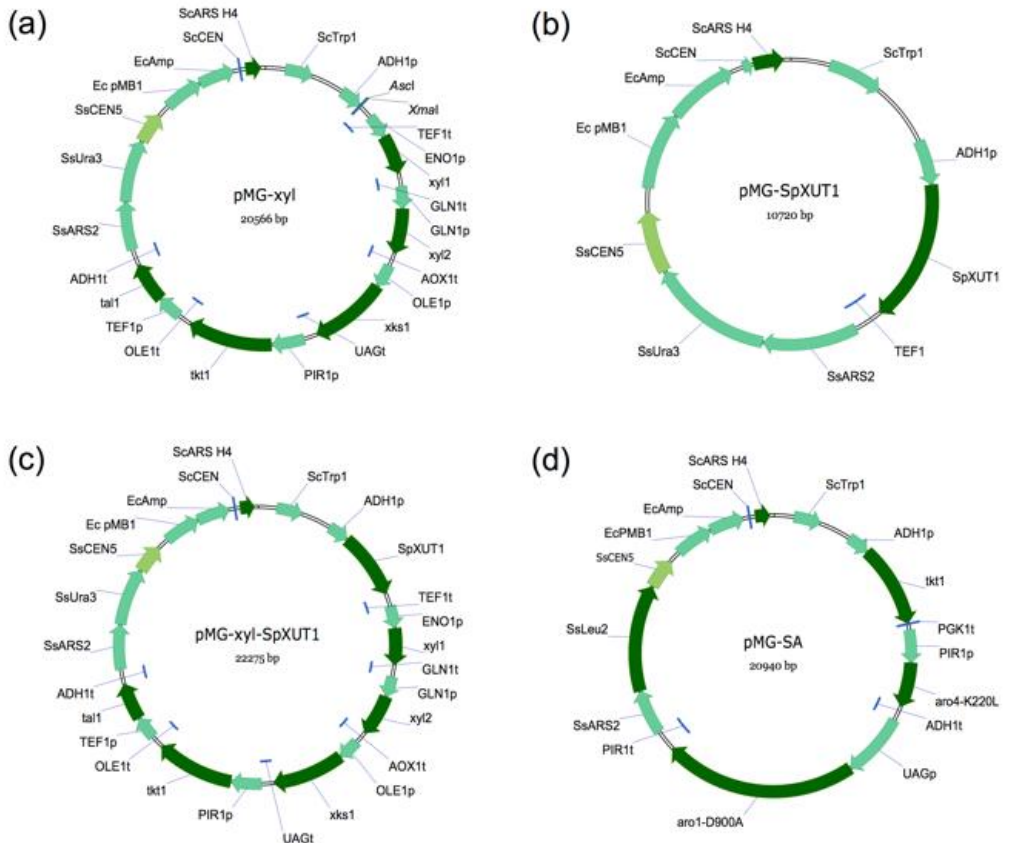

Supplementary Figure 2. The vector maps of the major plasmids constructed in this study. (a) pMG-xyl, (b) pMG-SpXUT1, (c) pMG-xyl-SpXUT1, and (d) pMG-SA4.

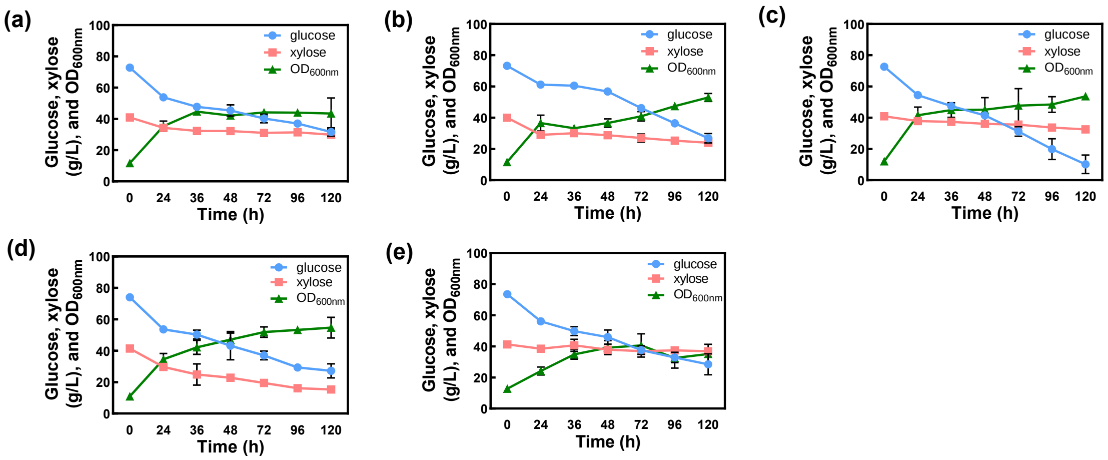

Supplementary Figure 3. The sugar utilization and cell growth profiles of the strain Ss-xyl expressing different xylose transporter genes in SC-ura plus 70 g/L glucose and 40 g/L xylose. (a) SsXut1, (b) SsXut2, (c) SsXut4, (d) SpXut1, or (e) no transporter in SC-ura plus 70 g/L glucose and 40 g/L xylose. Initial cell density was at ~10. The Ss-xyl expressing no transporter was used as a negative control strain. Ss, *Scheffersomyces stipitis*; Sp, *Spathaspora passalidarum*. Ss-xyl carries the engineered xylose assimilation pathway with the promoters of the five identified genes swapped with constitutive ones. Data are presented as mean ± S.D., $n = 3$ per group. It was interesting to observe that SsXut4 also led to a faster glucose consumption, suggesting its potential co-transport activity toward both glucose and xylose. Source data are provided as a Source Data file.

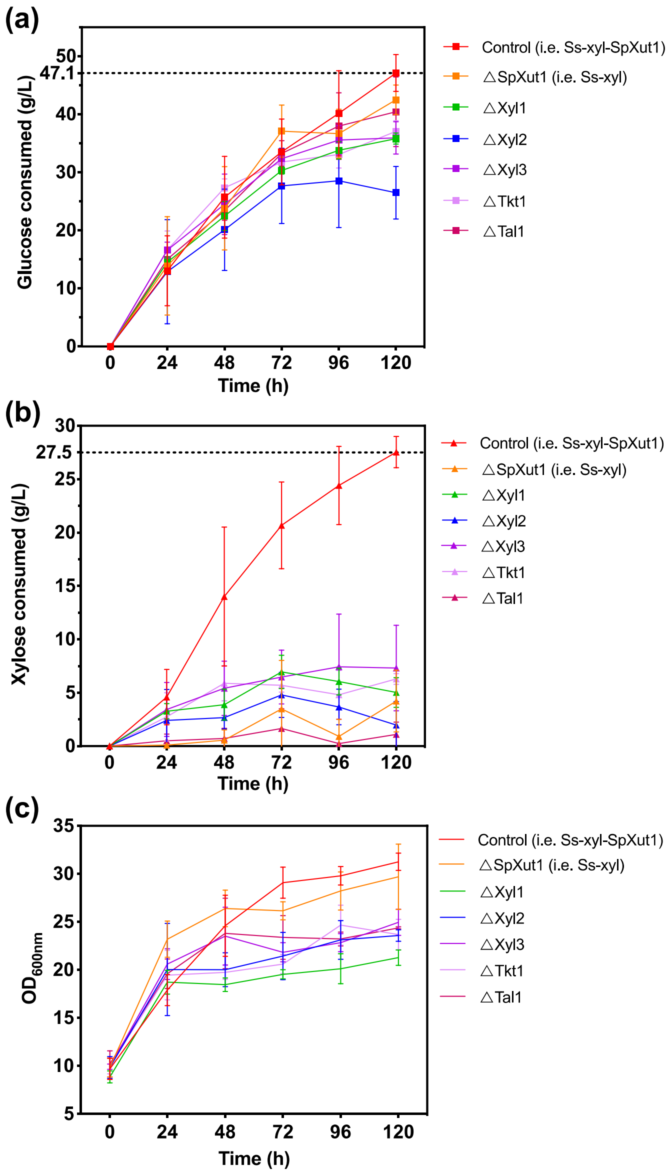

Supplementary Figure 4. The glucose consumption, xylose consumption, and cell growth profiles of the *S. stipitis* strains cultured in SC-ura medium containing 70 g/L glucose and 40 g/L xylose. Glucose consumption (a), xylose consumption (b), and cell growth (c) profiles of the *S. stipitis* strains cultured in SC-ura medium containing 70 g/L glucose and 40 g/L xylose. The derived variants were created by removing one gene at a time from the control strain Ss-xyl-SpXUT1 (i.e., the refactored xylose conversion pathway plus SpXut1 transporter). HCDF was conducted with an initial cell density of ~10. Samples were collected every 24 h. Data are presented as mean ± S.D., $n = 3$ per group. Source data are provided as a Source Data file.

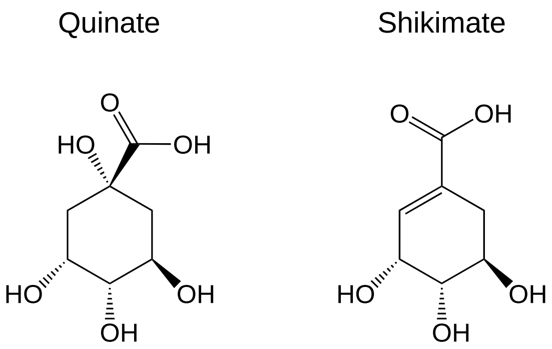

Supplementary Figure 5. The chemical structures of quinate and shikimate.

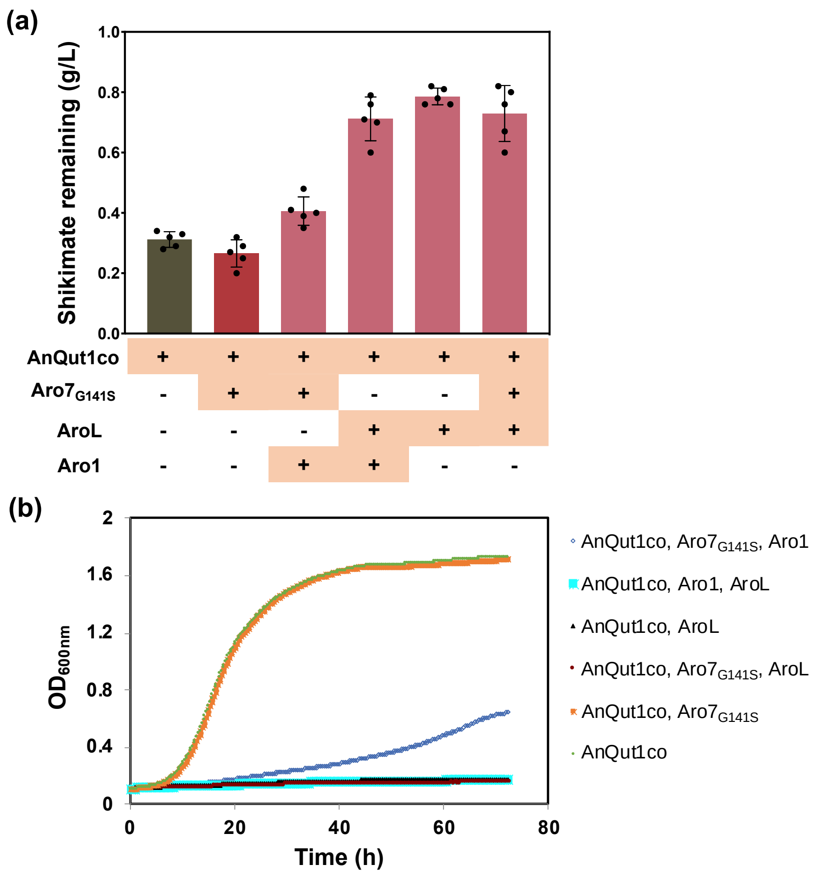

Supplementary Figure 6. The shikimate update and growth curves of the *S. cerevisiae* strains containing various combinations of the genes from the downstream pathway. (a) shikimate update, (b) growth curves. The growth curves of the *S. cerevisiae* strains overexpressing ScAro7$_{G141S}$, ScAro1, and/or EcAroL together with AnQut1co in SC-his-leu-ura media plus 40 g/L glucose and 1 g/L shikimate. Enzymes: AnQut1, quinate permease from *Aspergillus niger*; Aro7$_{G141S}$, feedback resistant chorismate mutase mutant; AroL, shikimate kinase; Aro1, penta-functional AROM protein. Data are presented as mean ± S.D., *n* = 5 per group. Source data are provided as a Source Data file.

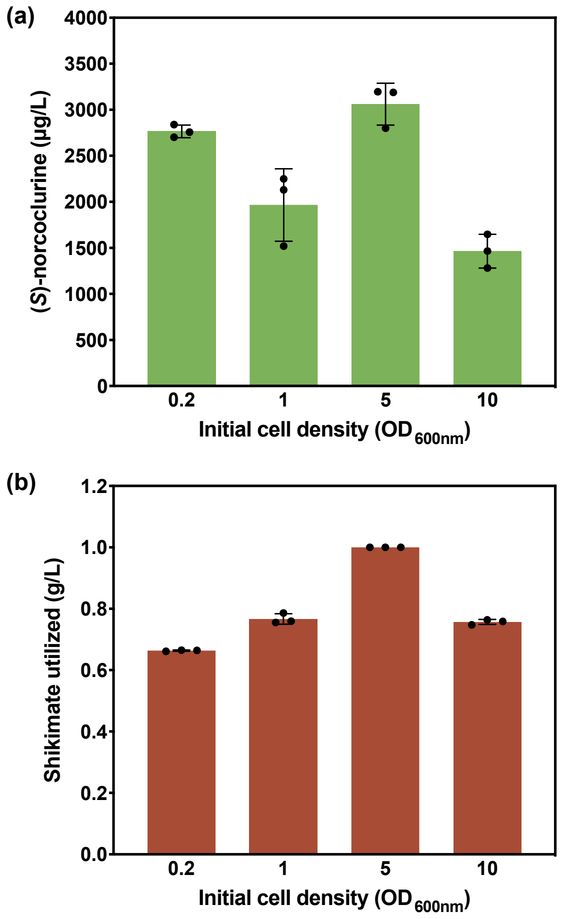

Supplementary Figure 7. (*S*)-norcoclaurine production and shikimate utilization by NC1 monocultures. (a) The titers of (*S*)-norcoclaurine yielded by different initial cell densities. (b) Shikimate utilization of the strain NC1 with different initial cell densities. NC1 was inoculated at different cell densities in 2xSC-his-leu-ura supplemented with 80 g/L glucose and 1 g/L shikimate. Samples were taken after four-day fermentation. Data are presented as mean ± S.D., $n = 3$ per group. Source data are provided as a Source Data file.

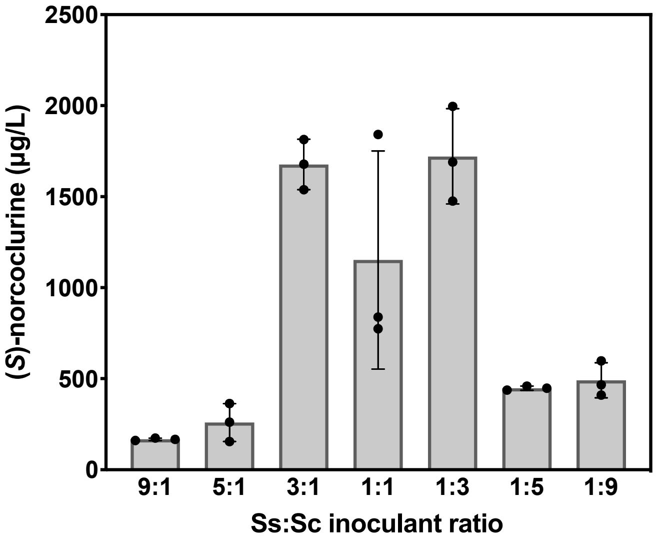

Supplementary Figure 8. (*S*)-norcoclaurine titers from the consortia with different initial Ss:Sc inoculating ratios. The initial total cell density (OD_{600}) was controlled at 0.2-0.3 and the two strains were simultaneously introduced into the culture. Samples were collected at 96 h. Data are presented as mean ± S.D., *n* = 3 per group. Source data are provided as a Source Data file.

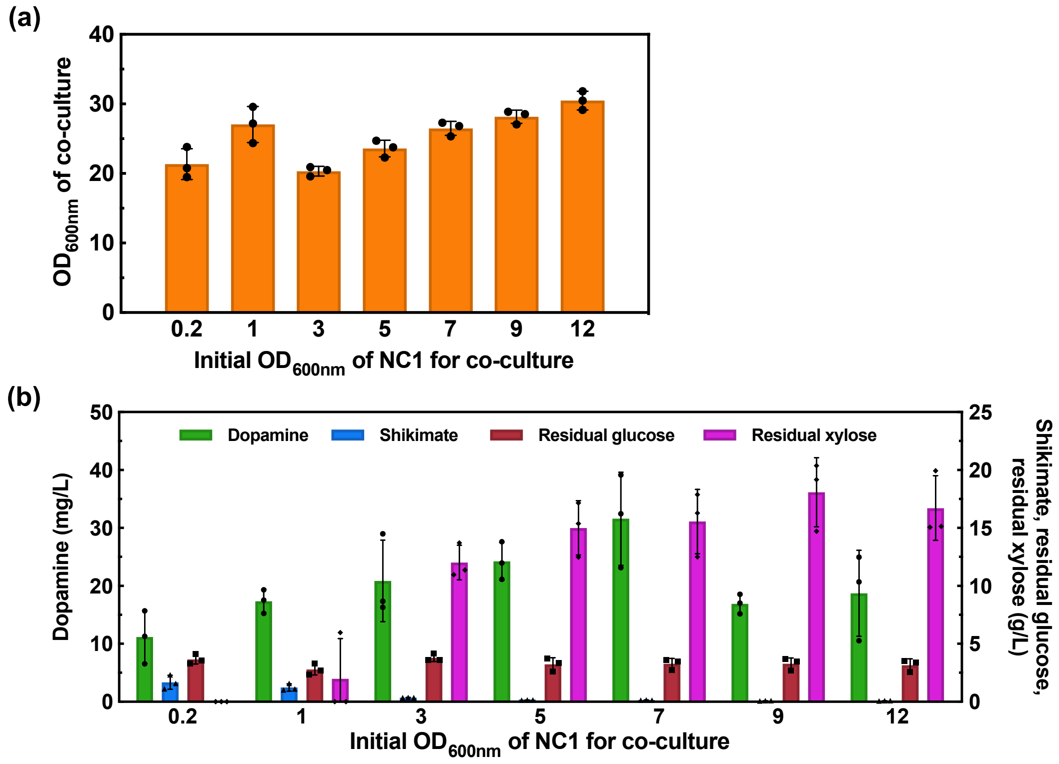

Supplementary Figure 9. Quantification of cell density, accumulation of dopamine and shikimate, and residual glucose and xylose in the cocultures consisting of SA4 and NC1 with various initial Ss:Sc inoculation ratios. The initial cell density of SA4 was 0.2-0.3 whereas the OD_{600nm} of NC1 was adjusted at different levels. The two strains were simultaneously introduced. Samples were collected after 96 h of fermentation. (a) Cell densities of the cocultures were measured at 600 nm after 96 h of fermentation. (b) Dopamine accumulation and residual shikimate, glucose, and xylose were monitored in the cocultures. Data are presented as mean ± S.D., *n* = 3 per group. Source data are provided as a Source Data file.

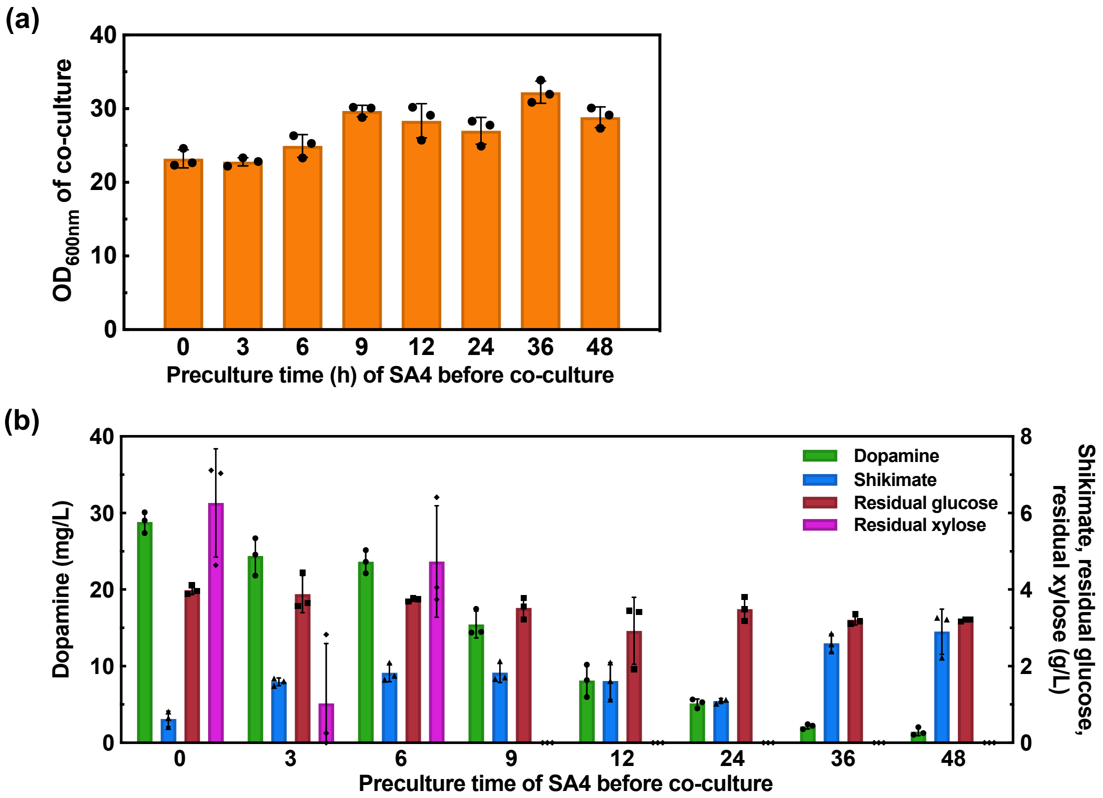

Supplementary Figure 10. Quantification of cell density, accumulation of dopamine and shikimate, and residual glucose and xylose in the cocultures consisting of SA4 and NC1 with a sequential inoculation strategy. *S. stipitis* SA4 was first grown in fermentation medium (2×SC medium containing 30 g/L glucose, 30 g/L xylose, and 5 g/L L-ascorbic acid) with an initial cell density of 0.2-0.3. After 0 h, 3 h, 6 h, 9 h, 12 h, 24 h, 36 h, and 48 h, *S. cerevisiae* NC1 was introduced into the corresponding *S. stipitis* SA4 culture with an OD_{600nm} of three. Samples were collected after 96 h of fermentation. (a) Cell densities of the cocultures were measured at 600 nm after 96 h of fermentation. (b) Dopamine accumulation and residual shikimate, glucose, and xylose were monitored in the cocultures. Data are presented as mean ± S.D., *n* = 3 per group. Source data are provided as a Source Data file.

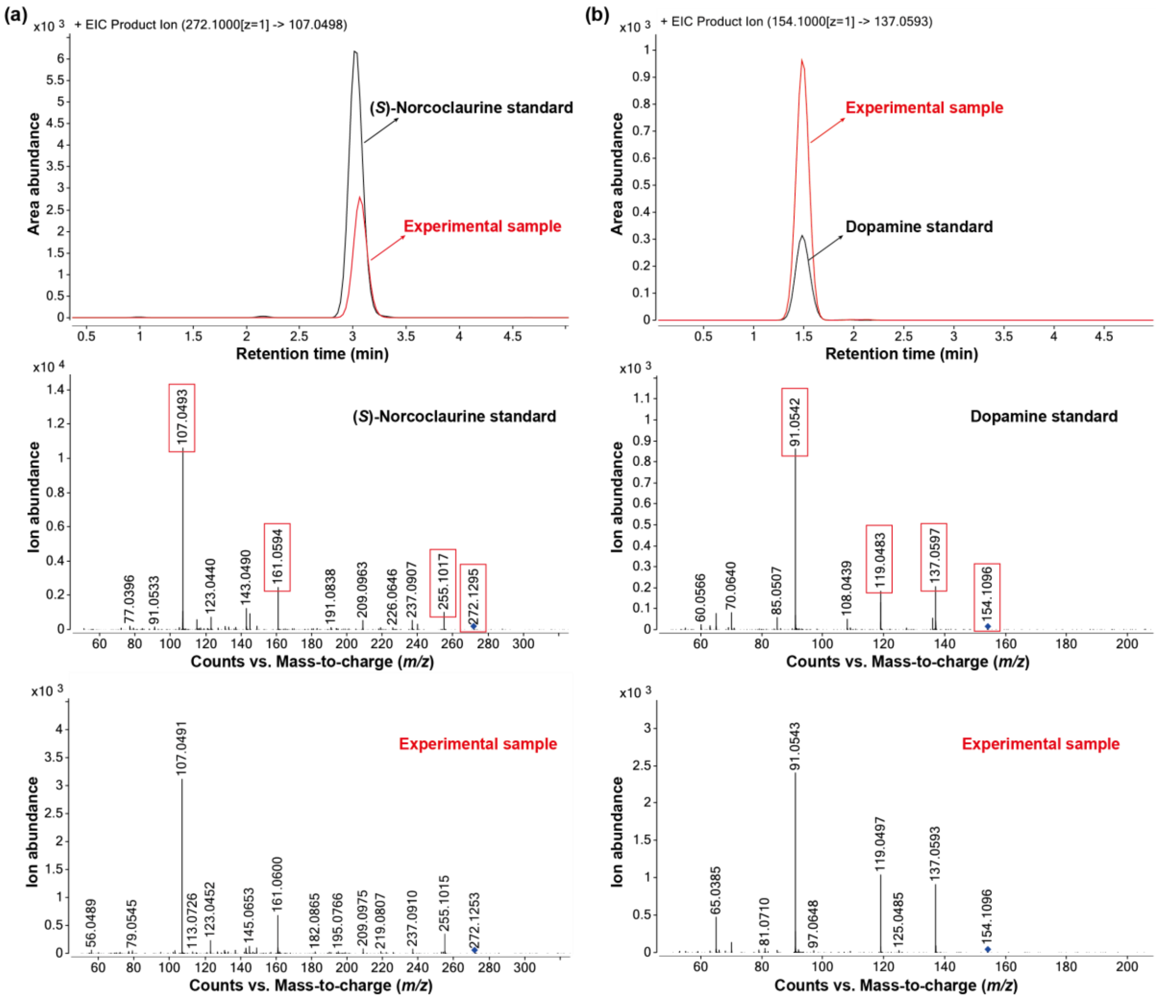

Supplementary Figure 11. LC-MS/MS analysis of (*S*)-norcoclaurine and dopamine in the supernatant of the SA4/NC1 co-culture after 72 h fermentation. (a) (*S*)-norcoclaurine and (b) dopamine. Fermentation was performed in 2×SC medium containing 30 g/L glucose, 30 g/L xylose, and 5 g/L L-ascorbic acid. Red boxes indicate the four signature $[M+H]^+$ ions of (*S*)-norcoclaurine standard ([*m/z*] 272.1, 255.1, 161.0, and 107.0) and dopamine standard ([*m/z*] 154.1, 137.0, 119.0, and 91.0).

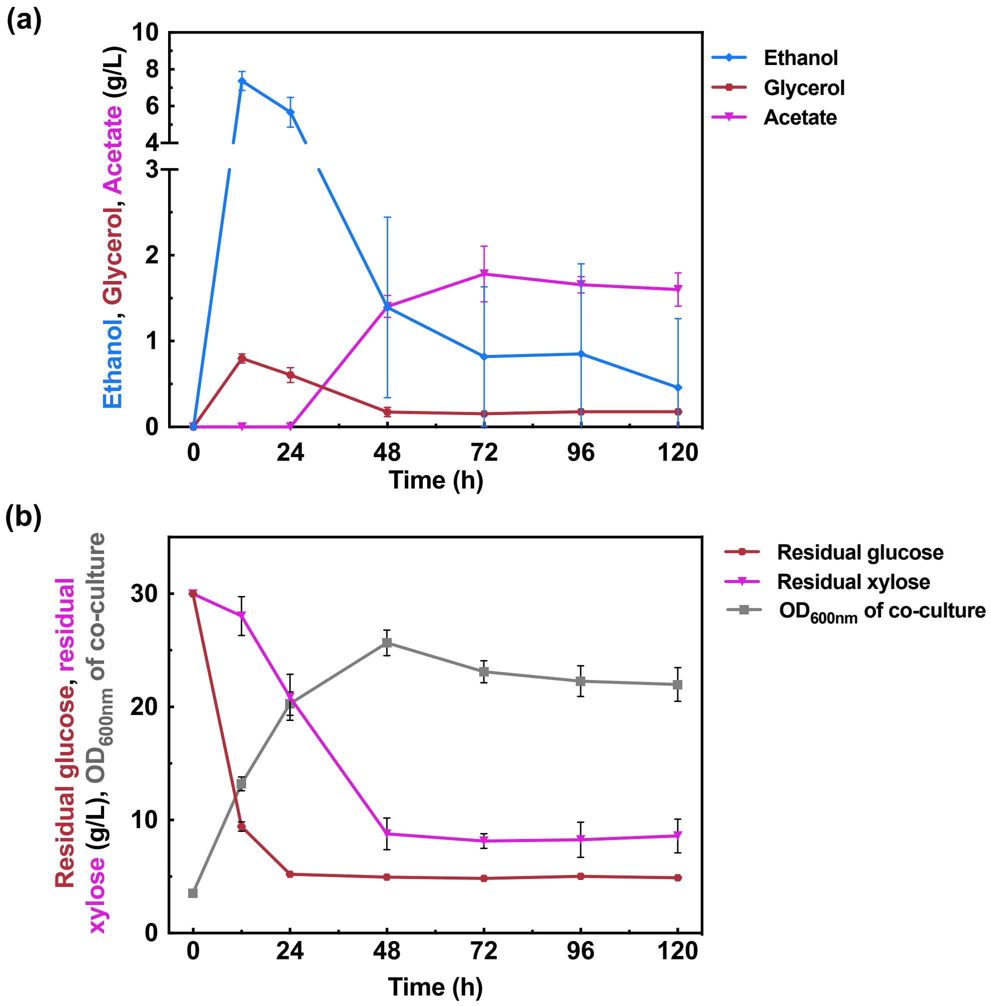

Supplementary Figure 12. Measurements of by-products (ethanol, glycerol, and acetate) and cell density (OD600nm) and residual glucose and xylose in the coculture consisting of SA4 and NC1 under the optimal co-culture condition. (a) by-products (ethanol, glycerol, and acetate) and (b) cell density (OD_{600nm}). The strains SA4 and the NC1 were simultaneously introduced into the culture with an OD_{600nm} of 0.2-0.3, and three, respectively. Samples were collected every 24 h post the starting of the co-culture. Data are presented as mean ± S.D., *n* = 5 per group. Source data are provided as a Source Data file.

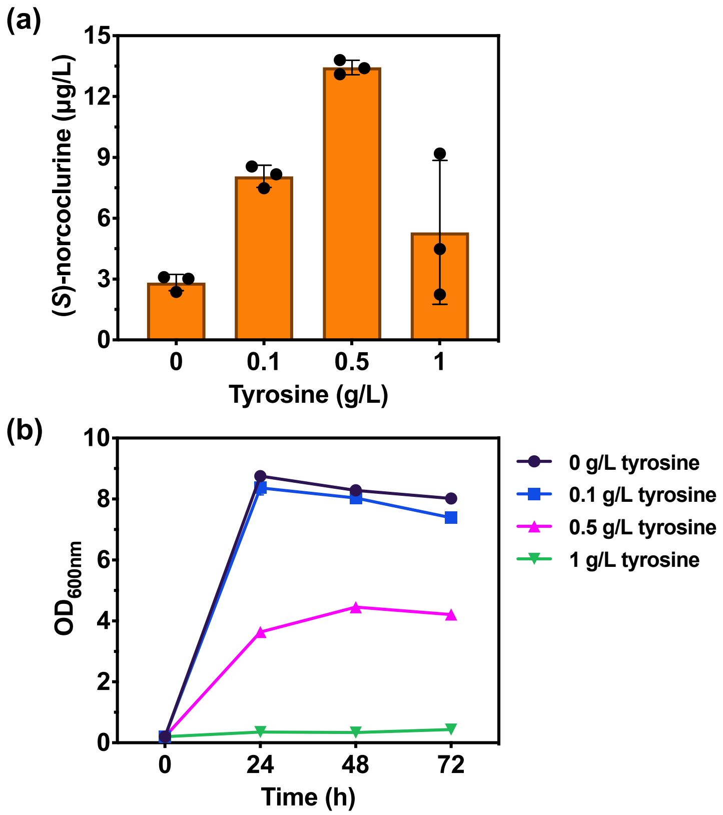

Supplementary Figure 13. (*S*)-Norcoclaurine production and cell growth profiles of the strain Sc-TDC fed with various concentrations of tyrosine in 72 h. (*S*)-Norcoclaurine production (a) and cell growth profiles (b). The recombinant *S. cerevisiae* strain Sc-TDC contains the plasmid pRS415-TyrH$_{W13LW369L}$-DODC-CjNCS. The initial OD$_{600nm}$ was set at ~0.2. The fermentation medium SC-leu was supplemented with different concentrations of tyrosine and 5 g/L L-ascorbic acid. Tyrosine stock at 50 g/L were prepared in 1N HCl. Samples were collected every 24 h. Data are presented as mean ± S.D., *n* = 3 per group. Source data are provided as a Source Data file.

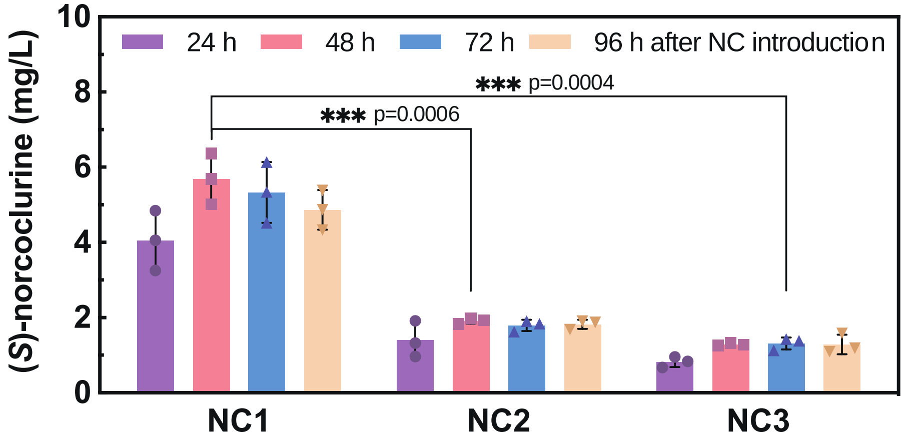

Supplementary Figure 14. (*S*)-norcoclurine production by yeast consortia with different copies of AnQut1co. After the strain SA4 was inoculated into fermentation medium with a starting OD_{600nm} of 0.2-0.3 and cultivated for 12 h, NC variants (NC1, NC2, and NC3) with OD_{600nm} of three was inoculated into the culture. Samples were collected every 24 h post the starting of co-culturing. Data are presented as mean ± S.D., *n* = 3 per group. Statistical analysis was performed using a two-sided Student's *t*-test. Selected comparisons are shown. ***$P < 0.001$. Source data are provided as a Source Data file.

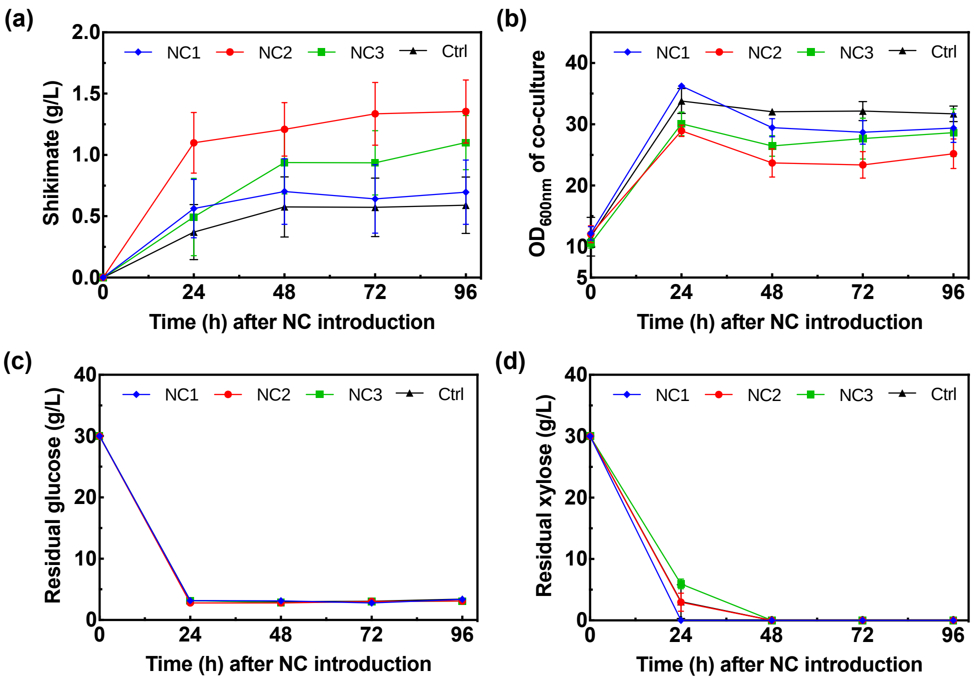

Supplementary Figure 15. Comparison of co-cultures including one, two, and three-copy of AnQut1co. Quantification of (a) shikimate accumulation, (b) OD_{600nm}, (c), residual glucose, and (d) residual xylose in the co-cultures consisting of SA4 and different NC variants (NC1, NC2 and NC3). After the strain SA4 was inoculated into fermentation medium with a starting OD_{600} of 0.2-0.3 and cultivated for 12 h, NC variants and the control (Sc-pRS356) with OD_{600nm} of three was inoculated into the culture. Samples were collected every 24 h post the starting of co-culturing. Data are presented as mean ± S.D., *n* = 3 per group. Source data are provided as a Source Data file.

Supplementary references

1. Gao MR, *et al.* Innovating a nonconventional yeast platform for producing shikimate as the building block of high-value aromatics. *Acs Synth Biol* **6**, 29-38 (2017).

2. Satoh K, Makimura K, Hasumi Y, Nishiyama Y, Uchida K, Yamaguchi H. *Candida auris* sp. nov., a novel ascomycetous yeast isolated from the external ear canal of an inpatient in a Japanese hospital. *Microbiol Immunol* **53**, 41 (2009).

3. Cadete RM, *et al. Spathaspora arborariae* sp. nov., a d-xylose-fermenting yeast species isolated from rotting wood in Brazil. *FEMS Yeast Res* **9**, 1338-1342 (2009).
