# Supplementary material for: Xylose and shikimate transporters facilitates microbial consortium as a chassis for benzylisoquinoline alkaloid production
Source: Nat Commun. 2023 Nov 28;14:7797. doi: 10.1038/s41467-023-43049-w (PMC10684500; doi:10.1038/s41467-023-43049-w)
Supplement: Supplementary file 3 — Description of Additional Supplementary Files [file 41467_2023_43049_MOESM3_ESM.pdf]

Description of Additional Supplementary Files

File name: Supplementary Data 1

Description: Strains and plasmids constructed in this study.

File name: Supplementary Data 2

Description: Protein sequences of multiple sugar transporters.

File name: Supplementary Data 3

Description: Protein sequence alignment of multiple sugar transporters. Ci, *Candida intermedia*; Sc, *Saccharomyces cerevisiae*; Ss, *Scheffersomyces stipitis*. The conserved motif G-G/F-XXX-G from CiGxs1 and the single point mutation in ScGAL2 and their counterparts in SpXut1 and SsXut were highlighted. The result was generated using Clustal Omega online (https://www.ebi.ac.uk/Tools/msa/clustalo/) with default parameters.

File name: Supplementary Data 4

Description: Primers used in this study.
