# Supplementary material for: Xylose and shikimate transporters facilitates microbial consortium as a chassis for benzylisoquinoline alkaloid production
Source: Nat Commun. 2023 Nov 28;14:7797. doi: 10.1038/s41467-023-43049-w (PMC10684500; doi:10.1038/s41467-023-43049-w)
Supplement: Supplementary file 6 — Supplementary Data 3 [file 41467_2023_43049_MOESM6_ESM.pdf]

```
CLUSTAL O(1.2.4) multiple sequence alignment


CiGXS1      MGLE------------------------------DNRMVKRFV---------------NV	15
ScGAL2      MAVEENNMPVVSQQPQAGEDVISSLSKDSHLSAQSQKYSNDELKAGESGSEGSQSVPIEI	60
SpXUT1      ---------------------MHGG-------SDGNDVQ-AIIAQKRLEIAGKPGIA---	28
SsXUT1      ---------------------MHGG-------GDGNDIT-EIIAARRLQIAGKSGVA---	28
SsXUT2      ---------------------------------------------------MKYFQI---	6
SsXUT3      -----------------------------------------MREVGILDVAHGNVVT---	16
SsXUT4      ------------------------------------------------------------	0
SsXUT5      -------------------------------------MT-------------ERSIGPLI	10
SsXUT6      ---------------------MSSV-------EKSAETASYTSQVSASGSAKTNSYL---	29
SsXUT7      ------------------------------------------------------------	0


CiGXS1      GEKKAGSTAMAIIVGLFAASGGVLFGYDTGTISGVMTMDYVLARYPSN----KHSFTADE	71
ScGAL2      PKKPMSEYVTVSLLCLCVAFGGFMFGWDTGTISGFVVQTDFLRRFGMKHKDGTHYLSNVR	120
SpXUT1      ---GLIANRKSFLIAVFASLGGLVYGYNQGMFGQISGMTSFSAAAGVG----KIQDNPTL	81
SsXUT1      ---GLVANSRSFFIAVFASLGGLVYGYNQGMFGQISGMYSFSKAIGVE----KIQDNPTL	81
SsXUT2      ---WKSGKQVSYAVTFTCELAFILFGIEQGIIGNLINNQDFLNTFGNP--------TGSY	55
SsXUT3      ---IMMKDPVVFLVILFASLGGLLFGYDQGVISGIVTMESFGAKF--P----RIFMDADY	67
SsXUT4      -------------------------------MSSLLTNEYFKDYYHNP--------TPVE	21
SsXUT5      PR---NKHLFYGSVLLMSIVHPTIMGYDSMMVGSILNLDAYVNYFHLTAATTGLNTAAVW	67
SsXUT6      ---GLRGHKLNFAVSCFAGVGFLLFGYDQGVMGSLLTLPSFENTFPAM----KASNNATL	82
SsXUT7      ------------------------------------------------------------	0


CiGXS1      SSLIVSILSVGTFFGALCAPFLNDTLGRRWCLILSALIVFNIGAILQVIST--AIPLLCA	129
ScGAL2      TGLIVAIFNIGCAFGGIILSKGGDMYGRKKGLSIV-VSVYIVGIIIQIASI-NKWYQYFI	178
SpXUT1      QGLLTSILELGAWVGVLMNGYVADRVGRRWSVMFG-VAWFILGVIIQACTHGANYSFILG	140
SsXUT1      QGLLTSILELGAWVGVLMNGYIADRLGRKKSVVVG-VFFFFIGVIVQAVARGGNYDYILG	140
SsXUT2      LGIIVSIYTLGCFFGCVMNFFIGDRMGRRSKIASS-MTVITIGVALQCSSF--SVEQLMI	112
SsXUT3      KGWFVSTFLLCAWFGSIINTPIVDRFGRRDSITIS-CVIFVIGSAFQC--AGINTSMLFG	124
SsXUT4      VGTMIAILEIGALFSSFIAGRVGDIVGRRRTIRYG-SFIFVVGGLVQATSV--NIVNLSL	78
SsXUT5      LGQVIATLT--------VISYFNDKFGRRSSVCIS-IAISLVGVALQSAAQ--NIEMFII	116
SsXUT6      QGAVIALYEIGCMSSSLATIYLGDRLGRLKIMFIG-CVIVCIGAALQASAF--TIAHLTV	139
SsXUT7      ----------------------------------------------MTFAV--NLYVFAV	12


CiGXS1      GRVIAGFGVGLISATIPLYQSETAPKWIRGAIVSCYQWAITIGLFLASCVNKGTEHMT--	187
ScGAL2      GRIISGLGVGGIAVLCPMLISEIAPKHLRGTLVSCYQLMITAGIFLGYCTNYGTKSYS--	236
SpXUT1      GRFIVGVGVGILSMIVPLYNAEVAPPEIRGSLVALQQLAITFGIMISYWITYGTNFIGGT	200
SsXUT1      GRFVVGIGVGILSMVVPLYNAEVSPPEIRGSLVALQQLAITFGIMISYWITYGTNYIGGT	200
SsXUT2      GRFITGLGTGWETSTCPMYQAELSPPKVRGRLVCSEALFVGVGLIYAYWFDYALSFTS--	170
SsXUT3      GRAVAGLAVGQLTMVVPMYMSELAPPSVRGGLVVIQQLSITIGIMISYWLDYGTHFIGGT	184
SsXUT4      GRLIAGIAIGFLTTIIPCYQSEISPPDDRGFYACLEFTGNIIGYASSIWVDYGFSFLD--	136
SsXUT5      GRIVIGFGISIGFVSSTILVSELAPPDKRGFILGLSFTSFLVGSLIAAGVTYGTRNAP--	174
SsXUT6      ARIITGLGTGFITSTVPVYQSECSPAKKRGQLIMMEGSLIALGIAISYWIDFGFYFLRND	199
SsXUT7      GRVLSGVGVGVLSTMVPSYQCEISPSEERGKLVCGEFTGNITGYALSVWADYFCYFIQDI	72
            .* : *.. .          .* :*   **            *   .

CiGXS1      ---------------------------NSGSYRIPLAIQCLWGLILGIGMIFLPETPRFW	220
ScGAL2      ---------------------------NSVQWRVPLGLCFAWSLFMIGALTLVPESPRYL	269
SpXUT1      G-----------------------EGQSKAAWLVPICIQMVPALILGSCIFLMPESPRWL	237
SsXUT1      G-----------------------SGQSKASWLVPICIQLVPALLLGVGIFFMPESPRWL	237
SsXUT2      ---------------------------GPIAWRLPLASQIVFAFVVFCFTFTIPESPRYM	203
SsXUT3      RCAPSHPYQGETFNPNVDVPPGGCYGQSDASWRIPFGVQIAPAVLLGIGMIFFPRSPRWL	244
SsXUT4      ---------------------------NDFSWRSPLYVQVVIGSMLFIGSFLIVETPRWL	169
SsXUT5      ---------------------------GDWCWRIPSIIQGAPDIVAIINILFISESPRWL	207
SsXUT6      G------------------------LHSSASWRAPIALQCVFAVLLISTVFFFPESPRWL	235
SsXUT7      GDAREKPH----------------SFFAHLSWRLPLFIQVVIAAVLFVGGFFIVESPRWL	116
                                           :  *         .       . .:**:

CiGXS1      ISKGNQEKAAESLARLRKL-PIDHPDSLEELRDITAAYEFETVYGKS---SWSQ------	270
```

```
ScGAL2      CEVNKVEDAKRSIAKSNKV-SPEDPAVQAELDLIMAGIEAEKLAGNA---SWGE------        319
SpXUT1      MNEGNEEKCLDVLSRLRGL-DRNNELIQMEFLEMKAQKIFEHELEAT--AYPDLQDGSAS        294
SsXUT1      MNEDREDECLSVLSNLRSL-SKEDTLVQMEFLEMKAQKLFERELSAK--YFPHLQDGSAK        294
SsXUT2      FYKGEKEEAKRILSYVFGK-PGDHPDILKEWNDINDAVILETSEG-A--FSWAK------        253
SsXUT3      LSKGRDEEAWSSLKYLRRK-SHED-QVEREFAEIKAEVVYEDKYKEK--RFPGKT-----        295
SsXUT4      LDHNHDIEGMIVISDLYADGDVEDDDAIAEYRNIKESVLIARV--EGGERSYQY------        221
SsXUT5      IAKERFSEAREIISIISDV-PIEDA--HEECEKIHAHIQTEKTAFPG--NKWK-------        255
SsXUT6      LNKGRTEEAREVFSALYDL-PADSEKISIQIEEIQAAIDLERQAGEG--FVLKE------        286
SsXUT7      LDVDQDQQGFHVLALLYD-SHLDDNKPREEFFMIKNSILLERETTPKSERTWKH------        169
                .  .    :         :      :   :

CiGXS1      -----VFS------HKNHQLKRLFTGVAIQAFQQLTGVNFIFYYGTTFFKRAGVNG-FTI        318
ScGAL2      -----LFS------TKTKVFQRLLMGVFVQMFQQLTGNNYFFYYGTVIFKSVGLDDSFET        368
SpXUT1      SRFKIGFLQYKSMLTHYPTFKRVAVACLIMTFQQWTGVNFILYYAPFIFASLGLSGKTTS        354
SsXUT1      SNFLIGFNQYKSMITHYPTFKRVAVACLIMTFQQWTGVNFILYYAPFIFSSLGLSGNTIS        354
SsXUT2      -----LFK-----PDKARTGYRVFLAYMSMFAQQLSGVNVVNYYITFVLINSVGIEDNLA        303
SsXUT3      -GVALTLTGYWDILTTKSHFKRVFIGSAVMFFQQFIGCNAIIYYAPTIFTQLGMNSTTTS        354
SsXUT4      -----LFTK---------YTKRLSVACFSQMFAQMNGINMVSYYAPMIFESAGWVGR-QA        266
SsXUT5      -----------QMVSSKSNTRRVIILFTQAIVTEMAGSSVGSYYFSIILTQAGVKDSNDR        304
SsXUT6      -----LFT-----QGPARNLQRVALSCWSQIMQQITGINIITYYAGTIFESYIGMSPFMS        336
SsXUT7      -----MFKN---------YMTRVLIACSALGFAQFNGINIISYYAPMVFEEAGFNNS-KA        214
                                 *:          :  * .   **   .:

CiGXS1      SLAT---NIVNVGSTIPGILLMEVLGRRNMLMGGATGMSLSQLIVAIVGVATS-------        368
ScGAL2      SIVI---GVVNFASTFFSLWTVENLGHRKCLLLGAATMMACMVIYASVGVTRLYPHGKSQ        425
SpXUT1      LLASGVVGIVMFLATIPAVLWVDQLGRKPVLISGALLMGMCHFVVAGILG----GLHGDF        410
SsXUT1      LLASGVVGIVMFLATIPAVLWVDRLGRKPVLISGAIIMGICHFVVAAILG----QFGGNF        410
SsXUT2      LILGGVAVICFTVGSLVPTFFADRMGRRLPSAVGAFGCGVCMMLISI-LL----SFQDNP        358
SsXUT3      LLGTGLYGIVNCLSTLPAVFLIDRCGRKTLLMAGAIGTFISLVIVGAIVG----KYGDRL        410
SsXUT4      ILMTGINSIIYIFSTIPPWYLVDSWGRKPLLLSGSVLMGVPLLTIACSLF----------        316
SsXUT5      LRVNIVMSSWSLVIALSGCLMFDRIGRKMQSLISLSGMIICFIVLGVLVK----EYGDGH        360
SsXUT6      RILAALNGTEYFLVSLIAFYTVERLGRRFLLFWGAIAMALVMAGLTV-TV----KLA--G        389
SsXUT7      LLMTGINSIVYWFSTIPPWFLVDHWGRKPILISGGLSMGICIGLIAVVIL----------        264
                          ::      :  *::     .

CiGXS1      ENNKSSQSVLVAFSCIFIAFFAATWGPCAWVVVGELFPLRTRAKSVSLCTASNWLWNWGI        428
ScGAL2      PSSKGAGNCMIVFTCFYIFCYATTWAPVAWVITAESFPLRVKSKCMALASASNWVWGFLI        485
SpXUT1      TNNMGAGWAAVVFIWLFAIFFGYSWGPCAWVIVAEVFPLGLRAKGVSIGASSNWLNNFAV        470
SsXUT1      VNHSGAGWVAVVFVWIFAIGFGYSWGPCAWVLVAEVFPLGLRAKGVSIGASSNWLNNFAV        470
SsXUT2      KLKKSSGAGAVAFFFVFQLVFGSTGNCIPWSMISELIPLHARAKGSSLATSSNWLWNFFV        418
SsXUT3      SEFKTAGRTAIAFIFIYDVNFSYSWAPIGWVLPSEIFPIGIRSNAISITTSSTWMNNFII        470
SsXUT4      LNNTYTPGVVVGSVIVFNAAFGYSWGPIPWLM-SEVFPNSVRSKGAAMSTATNWLFNFIV        375
SsXUT5      --SKSGSYAAVAMMFLFTGFYSFTFTPLNSLYPPELFPYVLRSTGVTLFNIFNGCWGLFA        418
SsXUT6      EGNTHAGVGAAVLLFAFNSFFGVSWLGGSWLLPPELLSLKLRAPGAALSTASNWAFNFMV        449
SsXUT7      LDKSFTPSMVAVLVIIYNASFGYSWGPIGFLIPPEVMPLAVRSKGVSISTATNWFANFVV        324
                            :   :. :          * :    ::   ::    .   .

CiGXS1      AYATPYMVDEDKGNLGSNVFFIWGGFNLACVFFAWYFIYETKGLSLEQVDELYEHVSKAW        488
ScGAL2      AFFTPFITSAINFYY----GYVFMGCLVAMFFYVFFFVPETKGLSLEEIQELWEEGVLPW        541
SpXUT1      AMSTPDFVAKATYGA----YIFLGLMCVFGAAYVFFFCPETKGRTLDEIDELFGDTSGVS        526
SsXUT1      AMSTPDFVAKAKFGA----YIFLGLMCIFGAAYVQFFCPETKGRTLEEIDELFGDTSGTS        526
SsXUT2      VEITPTIIEKLKWKA----YLIFMCCNFSFVPMFYFFFPETKNLTLEAIDDLFS------        468
SsXUT3      GLVTPHMLETMKWGT----YIFFAAFAIIAFFFTWLIIPETKGVPLEEMDAVFGDTAALQ        526
SsXUT4      GEMTPILLDTITWRT----YLIPATSCVLSFFAVGFLFPETKGLALEDMGSVFDDNSSIF        431
SsXUT5      SFILPIAMNGIGWKF----YIINACYDVIFLPIIMFCWIETKGINLDTISEVLHGRGPED        474
SsXUT6      VMITPVGFQSIGSYT----YLIFAAINLLMAPVIYFLYPETKGRSLEEMDIIFNQCPVWE        505
SsXUT7      GQMTPILQQRLGWGT----YLFPAGSCIISVIVVIFFYPETKGVELEDMDSVFESFY---        377
                *                .     .           ***.  *: :  :

CiGXS1      KSKGFVPSKHSFREQ----------VDQQMDSKTE-------AIMSEEASV---------        522
ScGAL2      KSEGWIPSSRRGNNY----------DLEDLQHDDKPW----YKAMLE-------------        574
SpXUT1      KREGEIRN-RILKE----VGLLELIGLEELDSDK---SKGGDVHYQEEKAADADADSA--        576
SsXUT1      KMEKEIHE-QKLKE----VGLLQLLGEENASESE---NSKADVYHVEK------------        566
SsXUT2      ------------------------------------------------------------        468
SsXUT3      EKNLV-----------------TITSVSESDAKD---RN--SIEMSE-------------        551
```

```
SsXUT4      SYHSTPSTGYGATESNSNARRASVISSENYQDSL--HQTAASLARNPSS-MRPDYDGIIT       488
SsXUT5      E-ESI-EESHSLIRQG------FV-----VNTKK-------------------------       495
SsXUT6      PWKVVQIA-RDLP----------IMHSEVLDHEKDVIIEKSRIEHVENIS----------       544
SsXUT7      NYKSPFKISRKRHQND----------GQAYQRV------ENDIRHNDVE-M-DDLDDLD-       418


CiGXS1      -----------------------------------------   522
ScGAL2      -----------------------------------------   574
SpXUT1      -----------------------------------------   576
SsXUT1      -----------------------------------------   566
SsXUT2      -----------------------------------------   468
SsXUT3      -----------------------------------------   551
SsXUT4      GAATLSPVPPLKPINISSNIPQEIEPPTFDEIFKYKLNEME   529
SsXUT5      -----------------------------------------   495
SsXUT6      -----------------------------------------   544
SsXUT7      -----------------------------------------   418
```

Protein sequence alignment of multiple sugar transporters. Ci, *Candida intermedia*; Sc, *Saccharomyces cerevisiae;* Ss, *Scheffersomyces stipitis*. The conserved motif G-G/F-XXX-G from CiGxs1 and the single point mutation in ScGAL2 and their counterparts in SpXut1 and SsXut were highlighted. The result was generated using Clustal Omega online (https://www.ebi.ac.uk/Tools/msa/clustalo/) with default parameters.
