# Supplementary material for: Xylose and shikimate transporters facilitates microbial consortium as a chassis for benzylisoquinoline alkaloid production
Source: Nat Commun. 2023 Nov 28;14:7797. doi: 10.1038/s41467-023-43049-w (PMC10684500; doi:10.1038/s41467-023-43049-w)
Supplement: Supplementary file 8 — Reporting Summary [file 41467_2023_43049_MOESM8_ESM.pdf]

nature portfolio

Corresponding author(s): Zengyi Shao

Last updated by author(s): Oct. 5th, 2023

Reporting Summary

Nature Portfolio wishes to improve the reproducibility of the work that we publish. This form provides structure for consistency and transparency in reporting. For further information on Nature Portfolio policies, see our Editorial Policies and the Editorial Policy Checklist.

Statistics

For all statistical analyses, confirm that the following items are present in the figure legend, table legend, main text, or Methods section.

n/a	Confirmed	
☐	☒	The exact sample size (*n*) for each experimental group/condition, given as a discrete number and unit of measurement
☐	☒	A statement on whether measurements were taken from distinct samples or whether the same sample was measured repeatedly
☐	☒	The statistical test(s) used AND whether they are one- or two-sided<br>*Only common tests should be described solely by name; describe more complex techniques in the Methods section.*
☒	☐	A description of all covariates tested
☒	☐	A description of any assumptions or corrections, such as tests of normality and adjustment for multiple comparisons
☐	☒	A full description of the statistical parameters including central tendency (e.g. means) or other basic estimates (e.g. regression coefficient) AND variation (e.g. standard deviation) or associated estimates of uncertainty (e.g. confidence intervals)
☐	☒	For null hypothesis testing, the test statistic (e.g. *F*, *t*, *r*) with confidence intervals, effect sizes, degrees of freedom and *P* value noted<br>*Give P values as exact values whenever suitable.*
☒	☐	For Bayesian analysis, information on the choice of priors and Markov chain Monte Carlo settings
☒	☐	For hierarchical and complex designs, identification of the appropriate level for tests and full reporting of outcomes
☒	☐	Estimates of effect sizes (e.g. Cohen's *d*, Pearson's *r*), indicating how they were calculated

Our web collection on statistics for biologists contains articles on many of the points above.

Software and code

Policy information about availability of computer code

Data collection	Image Lab 5.2.1 (BioRad)
Data analysis	Data analysis was performed using GraphPad Prism 9.3.1.<br>The MS/MS spectra data were analyzed using Agilent MassHunter Qualitative Analysis (version 10.0).

For manuscripts utilizing custom algorithms or software that are central to the research but not yet described in published literature, software must be made available to editors and reviewers. We strongly encourage code deposition in a community repository (e.g. GitHub). See the Nature Portfolio guidelines for submitting code & software for further information.

Data

Policy information about availability of data

All manuscripts must include a data availability statement. This statement should provide the following information, where applicable:

- Accession codes, unique identifiers, or web links for publicly available datasets
- A description of any restrictions on data availability
- For clinical datasets or third party data, please ensure that the statement adheres to our policy

All the data supporting the findings in this study is available within the Main Text and Supplementary Information. Source data is provided as a Source Data file. All the plasmids containing the refactored xylose pathway, the shikimate pathway, xylose transporters, shikimate transporters, and the norcoclaurine pathway that support the findings of this study are available from the corresponding author Zengyi Shao (zyshao@iastate.edu) upon reasonable requests.

Research involving human participants, their data, or biological material

Policy information about studies with human participants or human data. See also policy information about sex, gender (identity/presentation), and sexual orientation and race, ethnicity and racism.

Reporting on sex and gender	No information on sex and gender because there was no human research conducted.
Reporting on race, ethnicity, or other socially relevant groupings	No information on race, ethnicity, or other socially relevant groupings because there was no human research conducted.
Population characteristics	No information on population characteristics because there was no human research conducted.
Recruitment	No recruitment method was used because there was no human research conducted.
Ethics oversight	No ethics oversight was implemented because there was no human research conducted.

Note that full information on the approval of the study protocol must also be provided in the manuscript.

Field-specific reporting

Please select the one below that is the best fit for your research. If you are not sure, read the appropriate sections before making your selection.

- [x] Life sciences
- [ ] Behavioural & social sciences
- [ ] Ecological, evolutionary & environmental sciences

For a reference copy of the document with all sections, see nature.com/documents/nr-reporting-summary-flat.pdf

Life sciences study design

All studies must disclose on these points even when the disclosure is negative.

Sample size	Sample sizes, comprising a minimum of three biological replicates, were carefully chosen to align with our vast expertise and established protocols in yeast engineering. The selected sample sizes (N=3, N=4, or N=5) were not only based on our extensive experience but also maintained consistency with the throughput requirements of various experimental techniques. These techniques encompassed strain cultivation, measurement of sugar and metabolite concentrations, as well as quantification of cell densities.
Data exclusions	No data was excluded from the manuscript.
Replication	All experiments were conducted in biological triplicates, quadruplicates, or quintuplicates. A biological replicate in the context of our experimentation is one yeast colony streaked from the strain stock (not one individual culture from the same inoculum, as this represents a technical replicate). We confirmed that all attempts at replication were successful in this study.
Randomization	Randomization was not performed as the nature of the genetic and biochemical experiments and the associated data does not potentiate human bias influencing the final conclusions. In our particular study, we have focused on investigating the relationship between a specific independent variable (e.g., transporter, time, genotype, and initial OD) and the dependent variable (e.g., concentration and cell growth OD), without considering the influence of other variables. By simplifying the study design in this way, we aimed to gain a clearer understanding of the direct impact of the independent variable on the outcome, thus allowing for a more focused analysis and interpretation of the results.
Blinding	Blinding was not performed as the nature of the genetic and biochemical experiments and the associated data generated does not potentiate human bias influencing the final conclusions.

Reporting for specific materials, systems and methods

We require information from authors about some types of materials, experimental systems and methods used in many studies. Here, indicate whether each material, system or method listed is relevant to your study. If you are not sure if a list item applies to your research, read the appropriate section before selecting a response.

Materials & experimental systems

n/a	Involved in the study	
x		Antibodies
	x	Eukaryotic cell lines
x		Palaeontology and archaeology
x		Animals and other organisms
x		Clinical data
x		Dual use research of concern
x		Plants

Methods

n/a	Involved in the study	
x		ChIP-seq
x		Flow cytometry
x		MRI-based neuroimaging

Eukaryotic cell lines

Policy information about cell lines and Sex and Gender in Research

Cell line source(s)	S. cerevisiae YSG50 (requested from Dr. Huimin Zhao at UIUC)<br>S. stipitis FPL-UC7 (requested from Dr. Thomas W. Jeffries, professor emeritus at University of Wisconsin)<br>S. cerevisiae BY4741 (commercially available)<br>S. cerevisiae CEN.PK2-1C (commercially available)
Authentication	Cell lines were used without further authentication.
Mycoplasma contamination	Cells lines were not tested for mycoplasma contamination.
Commonly misidentified lines (See ICLAC register)	No commonly misidentified lines are included.
